# Enhancing the Bandwidth and Energy Production of Piezoelectric Energy Harvester Using Novel Multimode Bent Branched Beam Design for Human Motion Application

**DOI:** 10.3390/s23031372

**Published:** 2023-01-26

**Authors:** Iresha Erangani Piyarathna, Yee Yan Lim, Mahesh Edla, Ahmed Mostafa Thabet, Mustafa Ucgul, Charles Lemckert

**Affiliations:** 1 Faculty of Science and Engineering, Southern Cross University, Military Road, East Lismore, NSW 2480, Australia; 2 Sri Emas International School, Shah Alam, Selangor 40000, Malaysia; 3 Department of Research and Development, MYRA Corporate, UAG Bionutrients, Alstonville Industrial Area, Alstonville, NSW 2477, Australia; 4 Fortescue Future Industries Pty Ltd., 160 Lakes Rd, Hazelmere, WA 6055, Australia

**Keywords:** human motion, piezoelectric energy harvesting, bent branched beam harvester, macro-fiber composite (MFC)

## Abstract

In recent years, harvesting energy from ubiquitous ultralow-frequency vibration sources, such as biomechanical motions using piezoelectric materials to power wearable devices and wireless sensors (e.g., personalized assistive tools for monitoring human locomotion and physiological signals), has drawn considerable interest from the renewable energy research community. Conventional linear piezoelectric energy harvesters (PEHs) generally consist of a cantilever beam with a piezoelectric patch and a proof mass, and they are often inefficient in such practical applications due to their narrow operating bandwidth and low voltage generation. Multimodal harvesters with multiple resonances appear to be a viable solution, but most of the previously proposed designs are unsuitable for ultralow-frequency vibration. This study investigated a novel multimode design, which included a bent branched beam harvester (BBBH) to enhance PEHs’ bandwidth output voltage and output power for ultralow-frequency applications. The study was conducted using finite element method (FEM) analysis to optimize the geometrical design of the BBBH on the basis of the targeted frequency spectrum of human motion. The selected design was then experimentally studied using a mechanical shaker and human motion as excitation sources. The performance was also compared to the previously proposed V-shaped bent beam harvester (VBH) and conventional cantilever beam harvester (CBH) designs. The results prove that the proposed BBBH could harness considerably higher output voltages and power with lower idle time. Its operating bandwidth was also remarkably widened as it achieved three close resonances in the ultralow-frequency range. It was concluded that the proposed BBBH outperformed the conventional counterparts when used to harvest energy from ultralow-frequency sources, such as human motion.

## 1. Introduction

Over the past few decades, energy-harvesting systems have experienced remarkable growth due to the rapid emergence of the renewable energy industry. In addition to macroscale energy-harvesting technologies, such as solar and wind energy harvesting, innovations and the popularity of handheld electronic devices, medical devices, wireless sensors, and microelectromechanical (MEMS) systems have spurred the demand for micro-energy-harvesting techniques to minimize the reliance on conventional batteries. Using electrochemical batteries in devices such as intelligent electronics, smart wearables for biomedical monitoring, medical implants, wireless sensors, and other ultralow-power transferable electronics suffers from several shortcomings. This includes limited battery lifespan, practical difficulty with battery replacement, and environmental pollution arising from the disposal of batteries. In response to this, researchers have conducted numerous studies to explore alternative micro-energy-harvesting solutions to replace the need for conventional batteries or to extend the battery’s lifespan. Mechanical motion, particularly vibration or random displacements, is abundant and ubiquitous, from manmade systems to human bodies. In recent years, mechanical motion has emerged as a core energy source and has been widely studied for micro-energy harvesting.

Some popular mechanical energy-based transduction mechanisms are electrostatic, electromagnetic, triboelectric, and piezoelectric transduction [1]. Among these variant technologies for vibration-dependent energy harvesting, the piezoelectric transduction mechanism has attracted significant interest owing to its simplicity in design and implementation, good scalability, high power density, and minimal damping [2]. Moreover, piezoelectric devices can be manufactured on a microscale in flexible and stretchable miniature devices, marking a significant milestone in powering implantable medical devices [3]. The conventional linear piezoelectric energy harvester (PEH) is generally designed in unimorph or bimorph straight cantilevers, consisting of one or two piezoelectric transducers, respectively, often bonded on a metallic cantilever beam [4]. These piezoelectric patches are usually attached to the fixed end where the highest strain occurs. A proof mass is often attached at the free end to tune the system’s resonance frequency and enhance the vibration-induced stress [4].

Various studies on mechanical design improvement of piezoelectric energy-harvesting systems associated with high and low-frequency vibrations have been conducted [1,5,6,7,8]. Moreover, there have been recent developments in electrical design improvements, material improvements, and hybrid structural improvements with related analytical and theoretical frameworks of PEHs [9,10,11,12,13]. However, studies focusing on the ultralow excitation frequency [14] (i.e., 1 Hz to 10 Hz), such as human-induced motions, are relatively limited. The main reason is the inherent high natural frequency response of most conventional linear PEH systems, which significantly limits the effective energy harvesting from ultralow-frequency excitations [15].

The energy of ambient vibrations widely exists in nature and is often randomly distributed over a broad frequency spectrum. However, conventional PEH devices are usually designed to operate at their first resonance frequency within a narrow bandwidth. It is well understood that the PEH generates the highest output power when the entire system, or specifically the cantilever beam, is excited at its natural frequency. These have been identified as significant drawbacks of the device. Various inventive solutions have been proposed to widen the operating bandwidth, including tunable linear and nonlinear harvesters, impact-driven harvesters, cantilever arrays, and multimodal systems which possess multiple resonances across a broader frequency spectrum [16].

Multimodal PEH designs are relatively advanced systems with more than one proof mass that can achieve two or more close resonance peaks, resulting in a wider operating bandwidth [1]. Wu et al. [17] proposed a two-degree-of-freedom PEH consisting of inner and outer unimorph beams. To obtain multiple resonances, the weight of the tip mass was altered accordingly. Another widely used concept to achieve multiple close resonance peaks is PEH with multiple branched beams. Branches attached to the main beam can be tuned to achieve several close resonance peaks, leading to a widening operating bandwidth. Zhang and Hu [18] studied a PEH with several branched beams attached to the main beam with a single piezoelectric transducer. Tip masses were attached to each branched beam to increase the vertical deflection of the main harvester beam. Upadrashta et al. [19] continued experimenting with the branched beam concept and introduced a PEH capable of performing in a wider operating bandwidth of 15 to 20 Hz. The harvester comprises three sub-beams with various sizes attached to a main unimorph beam. Following their study, Izadgoshasb et al. [16] proposed a multi-resonance PEH by optimizing the beam shape, which was a unimorph beam with two triangular branches. Most of these designs were superior in terms of power density to the conventional linear PEH due to the ability of a single transducer to attain multiple resonance peaks. However, these energy harvesters are less effective when operating under multidirectional ambient vibration sources with a broader spectrum [20], such as those induced by human motion.

To tackle this problem, multidirectional harvesters with multiple resonances were introduced by a few researchers. Erturk et al. [21] proposed an L-shaped multidirectional design harvester with two close resonance peaks. Su et al. [22] further modified this L-shaped design into a V-shaped bent beam harvester and investigated its performance with varying angles. However, the output power generated was much smaller and, thus, not suitable to act as a standalone harvester for real-life applications. Zhao et al. [20] took another step to improve Su et al.’s design. With the aid of the distributed parameter model, they investigated the multimodal behavior of the V-shaped bent beam harvester. Two close resonance peaks were achieved within the 0 Hz to 60 Hz frequency spectrum, which considerably improved the output power. However, the design produced only a single resonance below 10 Hz when the angle was fixed at 45°. Since the design needed to be manually tuned, its practical applicability was deemed limited and unsuitable for ultralow-frequency vibration sources.

Further investigation was carried out by Jiang et al. [23] by introducing an impact stopper to the V-shaped bent beam harvester. Compared to other designs, the design seems to be improved with a broader operating bandwidth in low-frequency regions. Nevertheless, the lowest resonance peak lay between 8 Hz to 15 Hz, which was much higher than the frequency of human motion (1 Hz to 5 Hz). Furthermore, the power density of the harvester was low since the harvester consisted of two bimorphs, a connector, an impact stopper, and a proof mass in total. In addition, several design criteria must be addressed while downscaling the harvester for practical applications.

Several research groups have attempted different technical approaches to design PEH with multiple resonance peaks in a low-frequency spectrum to widen the operating bandwidth as discussed above [16,17,18,19,20,21,22,23]. However, it was found that it is very challenging to produce a design that achieves improvements in both power efficiency and bandwidth widening, particularly for a complex vibration source such as human motion. Moreover, a simple, standalone PEH design with fewer accessories is preferred considering the end-user’s comfort (human). Many preceding designs could hardly be miniaturized into microscale due to their complex design features, which are essential for wearable devices.

This study proposed a simple and effective multimodal PEH design, incorporating the V-shaped bent beam harvester and branched beam harvester for harvesting energy from ultralow-frequency excitation sources, particularly human motion. The key objective of this design was to improve the output voltage and power of the multimodal PEH merely through geometrical improvement, without the need for additional accessories (i.e., no weight penalty). The other objective was to broaden the operating bandwidth by achieving two or more close resonance peaks in the ultralow-frequency range.

The proposed multimodal PEH was designed to produce high power density using the branched beam concept. The designed bent branched beam harvester (BBBH) consisted of a unimorph beam with two bent branches attached at the free end. Finite element method (FEM) analysis was conducted to optimize the geometrical parameters of the BBBH based on the targeted frequency spectrum of human motion, while experimental tests were conducted using a mechanical shaker and human motion as sources of ultralow-frequency excitation. The performance of the proposed BBBH, in terms of the output voltage, the output power, the idle time, and the operating bandwidth, was compared with the existing designs, namely, the conventional cantilever beam harvester (herein denoted as CBH) and the V-shaped bent beam harvester (herein denoted as VBH). It is worth noting that a theoretical solution can be obtained by combining theoretical and FEA (including piezoelectric elements and electromechanical coupling) studies. However, this paper is focused on an experimental study in order to prove the proposed concept; hence, the theoretical study will be conducted in future research instead of in this study.

## 2. Materials and Methods

In this section, the materials and approaches used in the study are discussed.

### 2.1. Beam Harvesters Used in the Study

**Figure 1:**
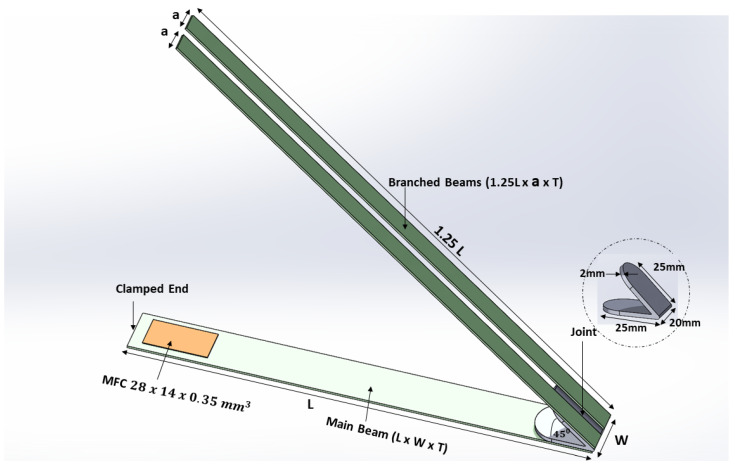
Schematic of proposed BBBH.

**Figure 2:**
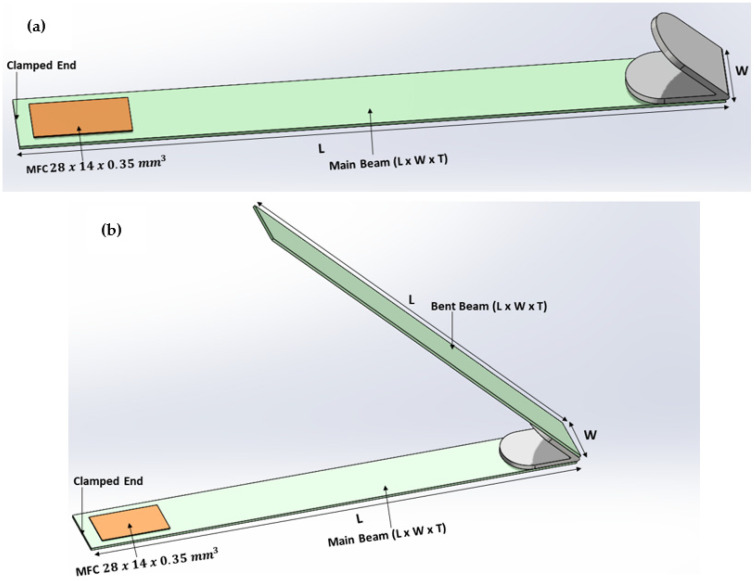
Schematics of two PEH designs; (**a**) CBH and (**b**) VBH.

The proposed BBBH consisted of one main cantilever beam and two branches folded in a V shape at 45° (Figure 1). A macro-fiber composite (MFC; M2814-P2) patch of 28 mm × 14 mm × 0.350 mm was attached near the fixed end of the main cantilever beam, and the two branches were attached to the other end through a joint. This study also considered two different beam designs, the CBH (Figure 2a) and the VBH (Figure 2b).

For comparison, the size and properties of the main cantilever beam (horizontal portion) were the same for all three designs. CBH consisted only of the main cantilever beam and the joint, acting as the proof mass. As for the VBH, the main cantilever beam was equivalent to the CBH, with a bent beam added at the free end through the joint, forming a V shape. Aluminum alloy (Grade 6063-T5) was used for various components of the beams, whereas stainless steel (Grade 304) was used for the joints. The overall volume of the proposed BBBH and the VBH were kept identical for a fair comparison.

### 2.2. Finite Element Method (FEM) Analysis

In order to understand the mechanical behavior of beam harvesters, FEM analysis was conducted using ANSYS Multiphysics [16,17,18,19] software. Three-dimensional (3D) hexahedral elements were used to model all the beams and the joints. This element contained 20 nodes, and each node possessed three translational degrees of freedom in the polar coordinate system. The element size selected was 1 mm, sufficiently acceptable to obtain accurate results while not incurring excessively long computational time [16].

The optimum dimensions of the main beam were then determined through a parametric study using the constructed FEM models. The main criterion is to achieve two or more close resonances in the ultralow-frequency range. Different geometries of the main cantilever beam were considered, directly affecting the vibratory response, such as the resonance frequency. Moreover, the suitability of the geometric properties for human motion application was also considered to achieve a user-friendly design.

Once the dimensions of the main cantilever beam were identified (denoted as L, W, and T standing for length, width, and thickness, respectively), it was used as the main beam (i.e., horizontal portion) for all three designs. For the VBH, the same dimensions (i.e., L, W, and T) were selected for the bent section. For the proposed BBBH, the length of the two branches was fixed at 1.25 L. This adjustment was made to ensure the total volume of BBBH was the same as VBH for a fair comparison. A gap was preserved between the two branches (width of each branch = a). Furthermore, 45° was adopted as the angle between the horizontal and the bent sections of the BBBH and the VBH, which was the optimal angle recommended in the previous study [20]. All symbolic dimensions are shown in Figure 1.

In the FEA, the main cantilever beams of all three designs were fixed at one end and free at the other to simulate the experimental condition. A V-shaped joint (weight: 15 g; size: 25 mm × 20 mm × 2 mm) was used as a proof mass for CBH and as a connector for VBH and BBBH. The material properties of the aluminum beam and the joint are summarized in Table 1. In addition, the material properties of the MFC actuator used in this study, are summarised in Table 2. These values have been obtained from the manufacturer’s profile (Smart Materials Corporation).

#### 2.2.1. Geometrical Optimization of Main Beam

Modal analysis was first conducted using FEM analysis to determine the optimum geometrical properties for the main cantilever beam with the first resonance frequency ranging between 1 Hz and 10 Hz. It is worth noting that during the FEM analysis, discretization and convergence criteria were considered to identify the most suitable number of nodes (12,200) and element size (1 mm) in the model. From this point onwards, there was no change in result accuracy by increasing the number of nodes in the system. When the number of nodes was greater than the current consideration (12,200<), the computational time required was excessively long. Hence, the element size was kept at 1 mm for all components of the model. The resonance frequencies and modal characteristics can be tuned by changing the main cantilever beam, including the length, thickness, width, and the attached proof mass. For this particular study, only the beam’s length and width were changed, while the proof mass and the thickness of the beam (1 mm) were kept constant. The minimum length considered in the parametric study was 75 mm, considering the length of the MFC patch (30 mm) and the length of the joint (25 mm).

For the first scenario, the width of the beam was kept at 20 mm, and the length was varied. Five trial lengths labeled Design L1 to L5 were considered (Table 3). The first six resonance frequencies (NF) of each length obtained from the modal analysis are presented in the table.

It can be observed that the first natural frequencies of Design L1 and Design L2 were much higher than the desired frequency range (i.e., <10 Hz) and, hence, were not suitable for this study. The first resonance of Design L3 was also considered too high for human motion. On the other hand, as stated previously, the volumes of the proposed BBBH and VBH were kept the same. For a main beam length of 250 mm (Design L5), the required branch length would be 315 mm for BBBH, which was relatively too long and undesirable. Therefore, Design L4 (L = 200 mm) was the most suitable design for this study.

To further tune the natural frequency according to the targeted frequency spectrum, the width of the beam was adjusted with the length fixed at 200 mm, based on Design L4. Considering that the width of the MFC was 15 mm, the minimum width for the trial was 20 mm. Three different widths (20 mm, 25 mm, and 30 mm) were analyzed, and the corresponding natural frequencies are presented in Table 4.

It was observed that the resonance frequency increased with increasing width. Note that the increase in width also remarkably affected the stress of the beam. For instance, when the width of the beam was 200 mm, the stress gain near the fixed end was 8.73 × 10^5^ Pa. The stress gain gradually decreases to 7.29 × 10^5^ Pa, and then 6.08 × 10^5^ Pa when the width is increased to 250 mm and then 300 mm, respectively. In addition, a lower natural frequency was preferred for human motion application; hence, the dimension of the main cantilever beam was selected as 200 mm × 20 mm × 1 mm for this study. For VBH, an identical section (200 mm × 20 mm × 1 mm) was attached at the free end of the main beam, forming an angle of 45° with the horizontal plane. As for BBBH, two identical branches (250 mm × 8 mm × 1 mm) were attached to the free end of the main beam at 45°. The gap between the two branches was 4 mm. Consequently, the volumes of VBH and BBBH were the same. However, it is worth noting that these dimensions were chosen as a proof of concept, and with the special characteristics of piezo and the simplicity of the device, it can be scalable to use as microstructures for real-time applications.

#### 2.2.2. Modal Analysis of CBH, VBH, and BBBH

Modal analysis was further conducted for all three PEH designs to identify their resonance frequencies in the ultralow range. The first six resonance frequencies of each PEH are presented in Table 5.

It can be noted that the CBH possessed only one resonance in the targeted frequency spectrum (<10 Hz). In addition, the first six resonance frequencies of the CBH spread over a wide frequency range of 5.39 Hz to 388.08 Hz. The VBH showed slightly improved behavior compared to the CBH, with a lower first resonance frequency of 4.50 Hz. Furthermore, the spread of natural frequencies was reduced from 4.50 Hz to 132.43 Hz. However, VBH also only possessed one resonance within the ultralow-frequency range.

The proposed BBBH, on the other hand, achieved a significant improvement compared to the CBH and the VBH. The first three resonance frequencies were 4.09 Hz, 8.72 Hz, and 10.56 Hz, indicating that the proposed BBBH could achieve three close resonance peaks in the targeted range (1 Hz–10 Hz), indicating the widening of operating bandwidth. This suggested that the proposed PEH could potentially harness higher output voltage and power than the other two designs in the frequency range of interest.

**Figure 3:**
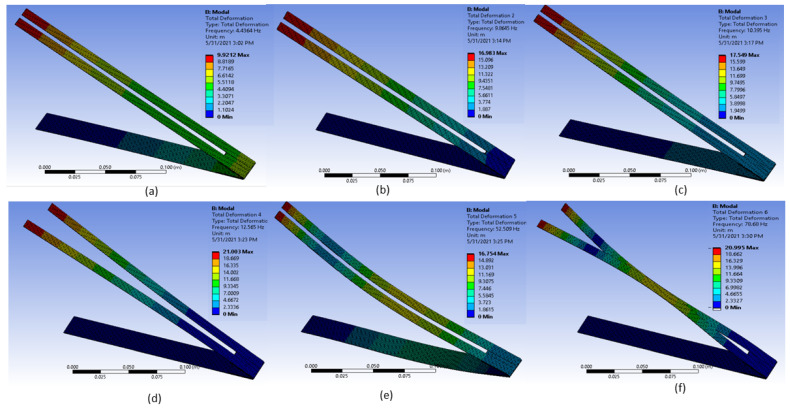
Deformed shapes of the first six resonances of BBBH obtained from modal analysis. (**a**) NF1, (**b**) NF2, (**c**) NF3, (**d**) NF4, (**e**) NF5, (**f**) and NF6.

The mode shapes and vertical displacements of the BBBH for the first six resonances were simulated as illustrated in Figure 3. It can be noted that both branches were vibrating in the same direction for the first, second, third, and fifth resonances which would generate maximum strain in MFC [19]. For the fourth and sixth modes, the branches were vibrating in opposite directions, which was expected to yield minimum strain in the MFC [19,24]. For this study, only the first three mode shapes were relevant since they fell within the desired frequency range.

#### 2.2.3. Static Analysis of CBH, VBH, and BBBH

The energy produced from PEH is directly related to the strain gained in the main cantilever beam. The study’s purpose of the static analysis was to elucidate the highest stress and strain achievable by various PEH designs. In the static analysis, the material properties were the same as those simulated for modal analysis. The bolt mass (1.2 g) was simulated as a point load [19]. The material properties of the beams and the proof mass (joint) are listed in Table 1. The simulation outcomes, in terms of highest strain and stress, are tabulated in Table 6.

As expected, the highest strain and stress of the cantilever beam occurred near the fixed end, where the piezoelectric patch was attached. It was noted that the CBH achieved the lowest stress of 8.73 × 10^5^ Pa, while the VBH attained a fairly similar stress level of 8.77 × 10^5^ Pa. The proposed BBBH yielded the highest stress of 9.74 × 10^5^ Pa, which was relatively higher than the other designs. A similar trend was observed with the induced strain. These results indicated that the proposed BBBH could generate relatively higher stress and strain, potentially generating higher voltage and power when dynamically excited.

### 2.3. Experimental Study

A series of experimental tests were conducted in two stages to study the performance of the proposed BBBH. In the first stage, the three designs were excited using a mechanical shaker to generate controlled and stable vibrations at the ultralow-frequency range. In the second stage, they were excited by human walking motions to generate real-life complex vibrations within the ultralow-frequency range. The performance of the three designs was studied and compared in terms of output voltage, output power, and idle time.

#### 2.3.1. Fabrication of the Harvesters

The size and design of the CBH, VBH, and BBBH were the same as the model developed in FEM analysis. All beam sections were fabricated using aluminum grade 6063-T5. For the CBH, a proof mass of 15 g was added at the free end of the main cantilever beam. The material properties of beams and joints are presented in Table 1. An MFC (M2814-P2) patch was attached near the fixed end of the main beam using two-part epoxy. The weight of the MFC patch was 0.5 g, and the size was 28 mm × 14 mm × 0.35 mm. However, it is worth noting that the size of the deployable energy harvester should be small; however, to demonstrate the concept and underlying working principle, a mesoscale-sized prototype was tested. With the special characteristics of piezo, the device can be scalable to use as microstructures for real-time applications.

#### 2.3.2. Stage 1: Shaker Test

**Figure 4:**
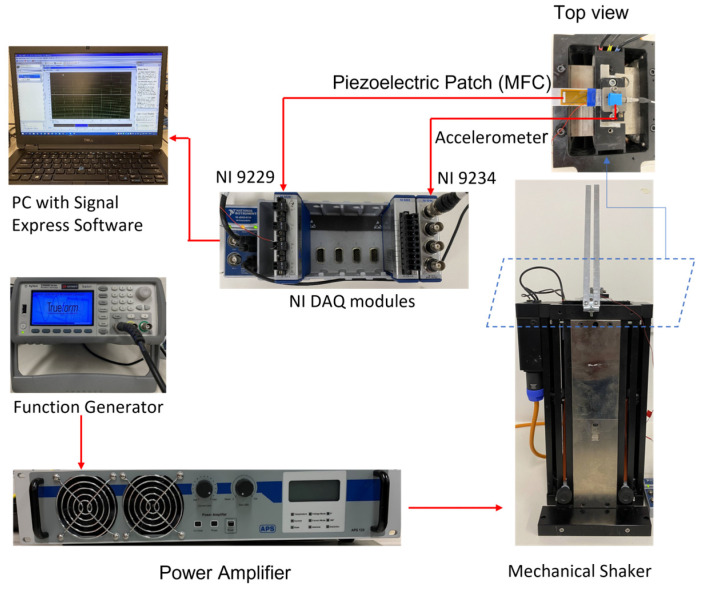
Experimental setup.

In Stage 1, all three PEH designs were tested with a mechanical shaker as the source of excitation. One end of the main cantilever beam was mounted on an electrodynamic shaker (APS-113, APS Dynamics, Inc. San Juan Capistrano, CA, USA) capable of emulating low-frequency vibrations. A power amplifier (APS125, APS Dynamics) was used to drive the mechanical shaker according to the input frequency from the function generator (Agilent 33210A). An accelerometer (Dytran 3305A2, 0.3 to 5000 Hz, ±5%) was fixed at the base of each PEH. The acceleration and the output voltage were measured using NI DAQ modules, NI 9234 and NI 9229, respectively, with the aid of Signal Express software. Next, the output voltage (V) was used to calculate the generated power under open circuit conditions. Considering that the load resistance (Rload) of the open circuit was close to infinity, a considerable load resistance (1 MΩ) [2,3] was assumed in the calculation. The output power was calculated as $V^{2}/R_{\mathrm{load}}$. The idle time of each harvester was then calculated by analyzing the duration of damping between each impact. A shorter duration of damping indicated a shorter idle time and, thus, high efficiency. Figure 4 illustrates the schematic of the experimental setup. Each PEH design was tested across a frequency range of 1 Hz to 5 Hz, at a step of 1 Hz. Two accelerations (i.e., 1 g and 2 g) were sequentially applied for each frequency.

#### 2.3.3. Stage 2: Human Motion Test

**Figure 5:**
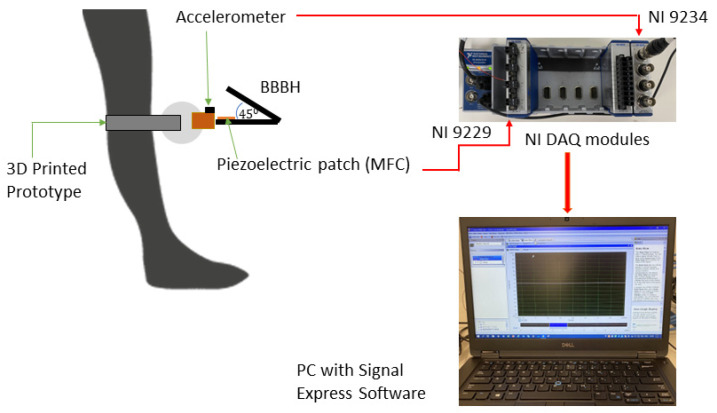
Schematic of experimental setup for the human motion test.

Human motion is a complex movement that can occur in various directions with different amplitudes and accelerations, depending on the source and intention of movements. In general, the amplitude is high, and the frequency is low. This study selected typical human motions with relatively steady and repetitive movements, namely, the walking and the running motions (as sources of excitation). A wearable, customized mechanical fixture [25] was used to attach the three PEH designs to the leg of a female weighing 54 kg, as illustrated in Figure 5. As explained previously in Section 2.3.1, the size of the proposed design can be easily downsized to fit into a wearable device, i.e., without harming the user [26]. Each PEH was attached to the mechanical fixture and tightened with screws to preserve the fixed end conditions. Previous studies found that the impact generated during foot strikes predominantly induced vertical acceleration [27,28,29,30]. Thus, for ease of comparison, the outcome was governed by vertical acceleration, which was considered the main motion with the largest amplitude.

It is worth noting that the mechanical fixture was affixed to the bony landmark location [31] to reduce the relative motion between the bone and the PEH system to minimize the damping effect caused by the skin [25]. The accelerometer was mounted at the base of the main cantilever beam to capture the acceleration induced by walking and running motions. Acceleration generated by different subjects could vary according to their weight, height, and habit of walking or running.

In order to obtain repeatable and stable outcomes, the test subject performed the walking and running motions on a treadmill at constant speeds of 2 km/h and 4 km/h, respectively. The frequencies measured were 1 Hz and 1.5 Hz, respectively. Table 7 gives a summary of all variables involved in the shaker test and the human motion test. In addition, the test subject was required to walk or run continuously for at least 5 min to reach a relatively stable state before data acquisition commenced. For each test scenario, three measurements were taken to ensure repeatability. Similarly, the acceleration and the output voltage were measured using the same NI DAQ modules. The test was repeated for all three designs.

## 3. Experimental Results and Discussions

Results obtained from mechanical shaker test and human motion test are discussed in this section.

### 3.1. Shaker Test

#### 3.1.1. Output Voltage

**Figure 6:**
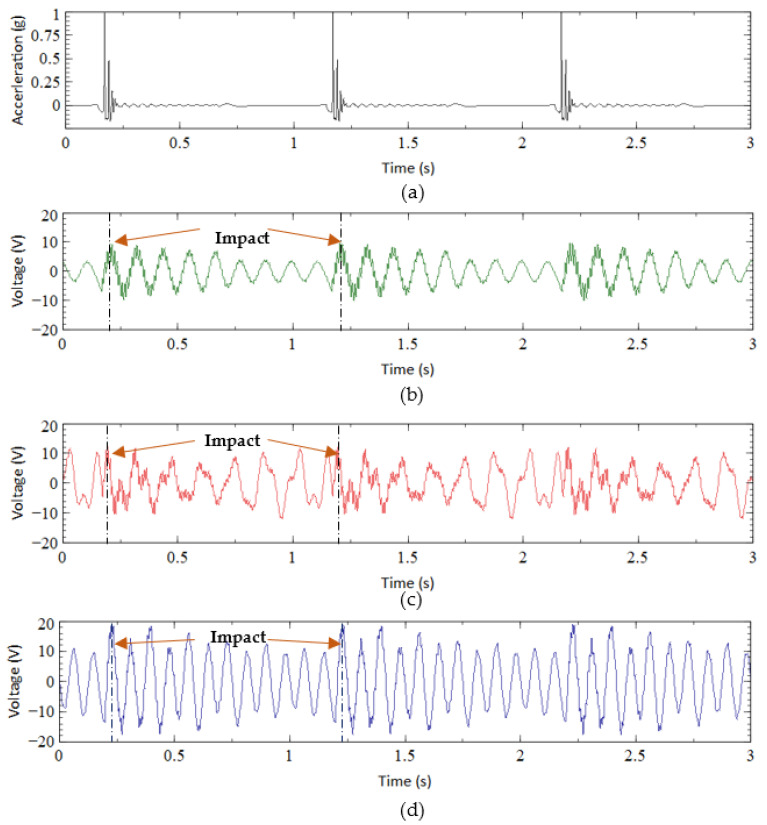
(**a**) Base acceleration and voltage output of (**b**) CBH, (**c**) VBH, and (**d**) BBBH under 1 Hz and 1 g acceleration.

In the shaker test, all three designs were subjected to two sets of base accelerations (1 g and 2 g) and five sets of frequencies (1, 2, 3, 4, and 5 Hz). Figure 6 illustrates the acceleration and output voltages against time measured from all designs under 1 Hz and 1 g base excitation. The highest output voltages obtained from CBH (Figure 5b) and VBH (Figure 5c) were about 10 V and 12 V, respectively. A slightly improved performance can be noticed in the VBH compared to the CBH. On the other hand, the voltage generated by BBBH (nearly 20 V in Figure 5d) was remarkably higher than the existing designs (i.e., CBH and VBH), which was approximately two times the CBH and one and a half times the VBH. This was deemed a significant improvement in output voltage for ultralow-frequency vibrations, achieved purely through geometrical design/modification, without additional components such as proof masses, magnets, shock vibrations, or impact stoppers.

**Figure 7:**
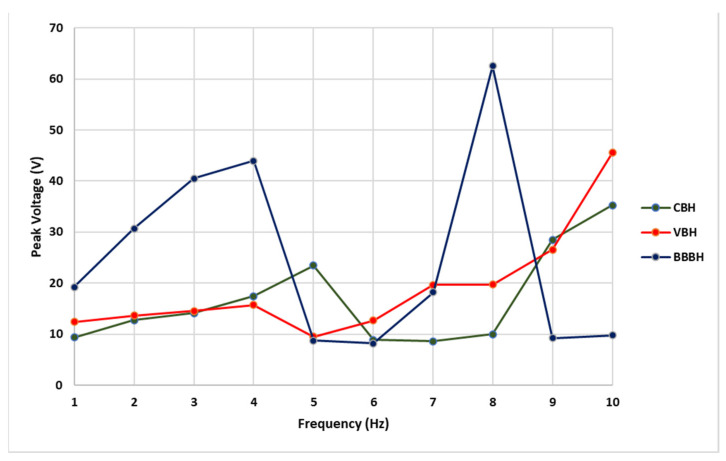
Output voltage of CBH, VBH, and BBBH under excitation frequency of 1 Hz–10 Hz under 1 g acceleration.

Next, the effect of varying excitation frequency was further investigated to understand the operating bandwidth of harvesters. For illustration, the peak output voltage recorded from all three designs under 1 g acceleration for 1 Hz to 10 Hz is presented in Figure 7. The results indicated that the proposed BBBH design was superior to the existing designs, producing the highest output voltage of 44 V at 4 Hz within the frequency range of interest (1 Hz–5 Hz). As per the FEM analysis, the first natural frequency of BBBH was 4.09 Hz. As expected, the higher output voltage was achieved when the excitation frequency was close to the natural frequency. Similarly, the highest voltage gained by VBH was 16 V at about 4 Hz, which was also close to the natural frequency obtained for VBH in the FEA analysis (i.e., 4.5 Hz). The CBH achieved its highest voltage of 23 V at about 5 Hz since the excitation frequency was close to its natural frequency (i.e., 5.39 Hz from FEA).

Another observation in Figure 7 was that the BBBH could perform remarkably at its second natural frequency, which was around 8 Hz (FEA 8.72 Hz), with a voltage output of 62 V. As noted in the FEM analysis study and experiments, CBH and VBH could achieve only one natural frequency within 1–10 Hz. Hence, this further proves that the proposed BBBH widened the operating bandwidth, achieving two natural frequencies below 10 Hz. The superiority of BBBH in voltage generation was due to the higher displacement caused by the amplification of the main cantilever beam by both branched beams.

Next, the same set of experimental tests was conducted for 1 Hz to 5 Hz under 2 g base acceleration. When the acceleration was increased to 2 g for 1 Hz, the general behavior of the three designs and the observations made were similar to the case of 1 Hz under 1 g acceleration. However, the magnitude of the voltage was higher due to higher acceleration. For instance, the BBBH was able to generate a maximum of 20 V for 1 Hz under 1 g acceleration. Under 2 g acceleration, BBBH produced 50 V, almost 2.5 times higher than the case of 1 g acceleration for 1 Hz. Considering that the acceleration was doubled, this outcome was expected. Similarly, for other frequencies (2 Hz to 5 Hz), a similar trend was also observed, although the magnitude of the voltage peak and the number of peaks per cycle were different. For simplicity, graphical representations of other cases were omitted.

#### 3.1.2. Idle Time 

The idle time can be used as an indicator of the efficiency of the PEH design. In this study, idle time referred to the period in which the energy harvested was relatively low. This usually occurs between each impact cycle and is particularly critical in the case of human motion, which is characterized by relatively large amplitude but a very low frequency. Generally, when the idle time is shorter, more spikes (i.e., voltage peaks) arbitrate in a motion cycle, resulting in higher output voltage and output power. If a PEH is not designed properly, the idle time is significant, resulting in low efficiency.

**Figure 8:**
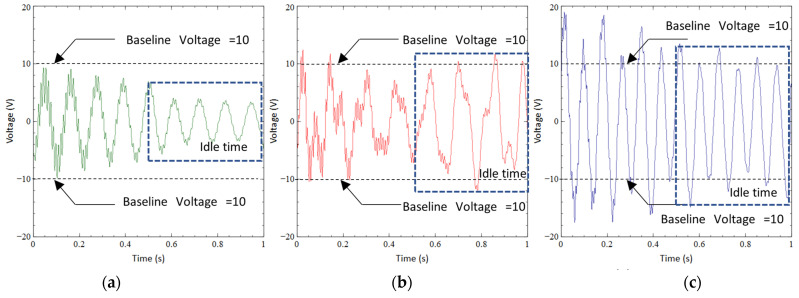
Idle time for (**a**) CBH, (**b**) VBH, and (**c**) BBBH at 1 Hz under 1 g acceleration.

In this study, the idle time of each harvester in a motion cycle was analyzed. In the case of CBH presented in Figure 8a, the peak voltage obtained was about 10 V during a single motion cycle (i.e., one second), representing a single impact given at the base of CBH by the mechanical shaker. Figure 6b illustrates that the peak voltage was achieved at the moment of impact. However, the voltage dramatically dropped immediately after the impact due to damping. Despite the spike in voltage within a short timeframe, approximately 0.5 s, or nearly half of the motion cycle, was idle or mainly wasted. For instance, if the peak voltage (10 V) of CBH at 1 Hz under 1 g acceleration was considered as the baseline. Approximately 0.5 s after the impact, the voltage generated reduced to 4 V, reflecting nearly 60% of the voltage drop. In the next half-motion cycle, the voltage further reduced to 2 V, reflecting nearly 80% of the voltage reduction compared to the baseline. This latter 0.5 s was referred to as the idle time of the CBH. During the idle time, the voltage generated was remarkably smaller than the peak voltage obtained. When the voltage was low, the average power or energy produced was also expected to be low. This is one of the major drawbacks of conventional CBH.

The peak voltage output of VBH was nearly 12 V (Figure 8b), which was a slight improvement compared to CBH. If the same baseline (peak voltage of CBH) was applied to VBH, the number of voltage spikes reaching or exceeding the baseline improved considerably. For instance, after 0.5 s of the impact (first half motion cycle) of VBH, the voltage reduced to 7 V, which was a 30% of voltage reduction compared to the baseline. During the next half motion cycle, VBH still produced voltage spikes of 10 V (a gain of 100% compared to the baseline). This can be due to the increased vertical displacement of the bent section of VBH, which amplifies the main beam’s vertical displacement. Compared to the CBH, the idle time of VBH was reduced since the voltage reduction of the whole motion cycle was only about 30%. Hence, VBH was capable of producing a higher voltage output with idle time in each motion cycle compared to CBH.

In the case of BBBH, the peak voltage generated by the harvester was about 20 V, which was two times higher than the peak voltage of CBH (the baseline voltage). If the same baseline was considered for BBBH, all the voltage spikes obtained for BBBH were equal to or larger than the peak voltage of CBH. To be further explicit, the whole motion cycle is presented in Figure 8c. The peak voltage of 20 V was generated at the impact instance, which was 200% of the baseline. Then, after 0.5 s, BBBH could still generate 16 V, which was 160% of the baseline voltage. During the next half-motion cycle, BBBH produced a minimum of 10 V equal to the baseline voltage (100%). Therefore, the voltage drop relative to the peak voltage generated by CBH was negligible for the case of BBBH. This reflected that the idle time of BBBH was remarkably improved compared to CBH and VBH. The reduction in idle time greatly enhanced the efficiency of the harvester.

#### 3.1.3. Number of Voltage Peaks/Spikes

It was noticed that the number of voltage spikes generated during one motion cycle varied across different harvesters. This number of voltage spikes, and their corresponding magnitude, was also a useful indicator of the performance of different harvesters. In general, more spikes indicate less idle time and, thus, higher efficiency. For such a purpose, the output voltage of CBH, VBH, and BBBH at 1 Hz under 1 g acceleration within one motion cycle was considered. Again, the peak output voltage of CBH (10 V) was considered as the baseline for the comparison. With such conditions, the CBH could only produce one 10 V voltage spike within one motion cycle. For the VBH, approximately six voltage spikes reached or exceeded the baseline (10 V) within 1 s, which was a considerable improvement.

On the other hand, the BBBH was able to produce 24 voltage spikes which were equal to or larger than the baseline voltage within one motion cycle, implicating a significant reduction in idle time. The presence of stronger voltage spikes was mainly due to the two branched beams, which amplified the main beam vibration due to the higher vertical displacement experienced by the branches in a single impact. The improvement of BBBH, in terms of the number of voltage spikes, was 24 times and 4 times more than CBH and VBH, respectively. Table 8 tabulates the baseline voltage and the number of peaks attained for all three designs under various excitation conditions. A similar trend was also observed for all the five frequencies under 2 g acceleration; however, for simplicity, representations of these cases were omitted.

#### 3.1.4. Harvested Power

**Figure 9:**
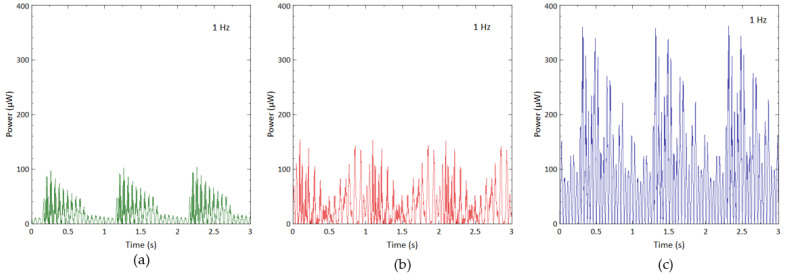
Output power of (**a**) CBH, (**b**) VBH, and (**c**) BBBH when excited under 1 Hz and 1 g acceleration.

The output power was calculated to further analyze the proposed design’s productivity. Because the open circuit’s load resistance (Rload) was close to infinity, a considerable 1 MΩ load resistance was assumed in the calculation [16,19]. The output power was calculated as $V^{2}/R_{\mathrm{load}}$. Figure 9 illustrates the power output by various designs during three motion cycles under the impact of 1 Hz from the mechanical shaker at 1 g acceleration. The output power from different designs varied remarkably, while their trends were similar to the output voltage. Figure 9a shows that the highest power generated by CBH was 90 μW. The power between each motion cycle decreased rapidly due to damping after each impact.

The power generated by the VBH was considerably higher than the CBH in one complete cycle, as presented in Figure 9b. The highest power of nearly 160 μW was achieved. However, similar to the case of voltage, the power remained relatively low between each impact. The power generated by BBBH is illustrated in Figure 9c. BBBH generated a maximum power of 360 μW, which was more than twice the VBH’s highest power. In addition, the BBBH could produce power ranging from 90 μW to 300 μW during each cycle. It is worth mentioning that the average low-power peaks generated by BBBH were higher than or equal to the highest power achieved by the CBH.

Thus, the BBBH was identified as the most efficient PEH in power generation among the three designs. This superiority was explained earlier in the study of output voltage, where the higher displacement caused the amplification of the main cantilever beam by both branched beams. As per the observations made during the experiment, the influence of multidirectional vibration experienced by the branches can be another cause for this high-power generation. Further study is recommended to understand better the interaction between the branches and the main beam, as well as the mechanisms involved in voltage and power amplification.

**Figure 10:**
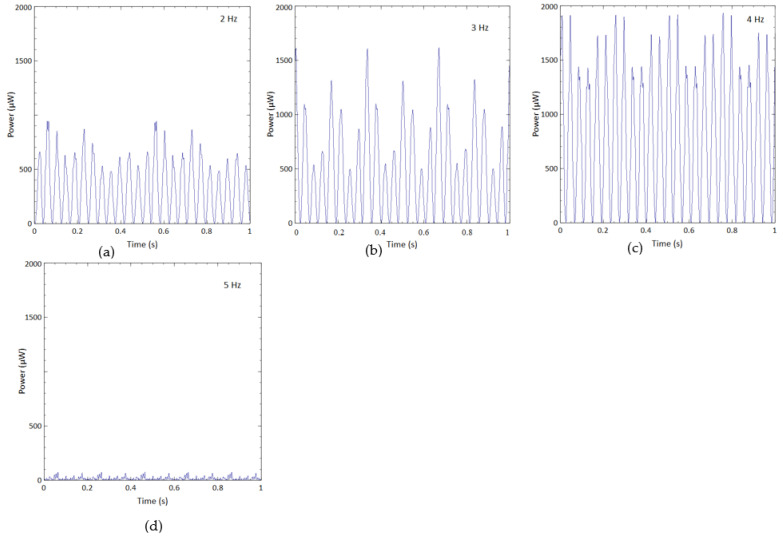
Output power generated by BBBH at (**a**) 2 Hz, (**b**) 3 Hz, (**c**) 4 Hz, and (**d**) 5 Hz under 1 g acceleration.

Graphical representations of other frequencies (2 Hz to 5 Hz) for BBBH under 1 g acceleration are provided in Figure 10. BBBH was capable of producing at least 500 μW for the cases of 2 Hz and 3 Hz. Overall, the optimum performance of the BBBH was observed at 4 Hz, producing power ranging from 1400 μW to 1900 μW. However, the output power at 5 Hz decreased dramatically as the excitation frequency (5 Hz) was away from the natural frequency.

The superior performance of BBBH over the counterparts was also observed in these frequency ranges, and the details were omitted herein for simplicity. A similar trend was also noticed for various excitation frequencies under 2 g acceleration.

#### 3.1.5. Average Output Power

**Figure 11:**
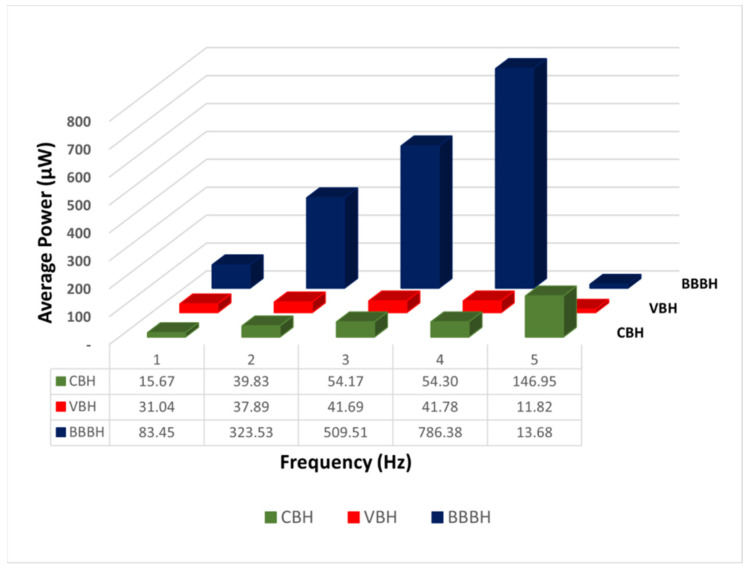
Comparison of average output power against frequency generated by three PEH designs under 1 g acceleration.

To further understand the power generation of each PEH design under varying acceleration and frequency, the average output power produced across one cycle was calculated and illustrated in Figure 11. The average output power generated from the CBH gradually increased from 15.67 μW to 146.95 μW when the frequency was increased from 1 Hz to 5 Hz. A slight improvement of 31.04 μW in average output power was noticed for the VBH at 1 Hz under 1 g acceleration compared to the CBH. For higher frequencies (2 Hz to 4 Hz), the average output power gradually increased from 37.89 μW to 41.78 μW; however, for these frequencies, the performance of VBH was similar to CBH. The lowest average power of 11.82 μW was obtained at 5 Hz for VBH when the excitation frequency was away from its natural frequency.

On the other hand, the BBBH showed a dramatic improvement in the average output power from 1 Hz to 4 Hz, with values ranging from 83.45 μW to 786.38 μW, respectively. The lowest average output power of BBBH, 13.68 μW, achieved at 5 Hz, coincided with the lowest average output power achieved by CBH at 1 Hz.

### 3.2. Human Motion Test 

The human motion test was further conducted to study the proposed design’s suitability in practical applications. Once again, the results obtained from the BBBH were compared against the CBH and the VBH. The output voltage and power produced during walking (1 Hz) and running (1.5 Hz) motions were recorded, analyzed, and discussed. Furthermore, the efficiency of the harvester in terms of idle time and the average output power was discussed.

#### 3.2.1. Output Voltage

**Figure 12:**
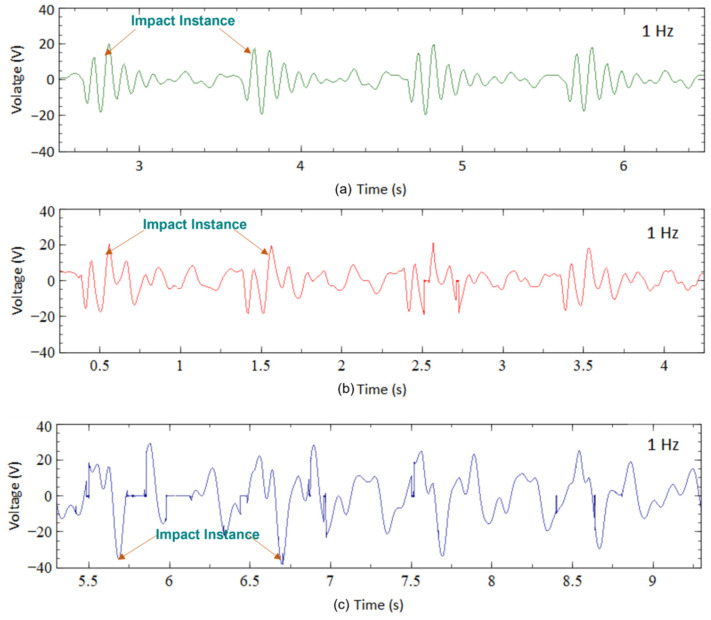
Voltage output of (**a**) CBH, (**b**) VBH, and (**c**) BBBH for human walking (1 Hz).

Figure 12 presents the output voltage generated by the three designs during walking with an impact frequency of 1 Hz. This impact occurred when the heel of the leg hit the ground. The acceleration of each impact was controlled at approximately 0.2 g for all designs. The highest output voltage generated by the CBH under such circumstances was about 20 V (Figure 12a) at the time of impact. Like the shaker test, the voltage decreased dramatically due to damping. Figure 12b represents the output voltage of VBH under the same excitation condition. The highest output voltage from VBH for walking motion was about 22 V, slightly improving than the CBH. This observation matched the trend observed in the mechanical shaker test. Figure 12c shows the output voltage generated by the BBBH during the walking motion. The BBBH produced nearly 40 V during the impact, with almost double the highest voltage produced by the CBH and the VBH. Several voltage peaks (10–30 V) were present between each impact, while the period of most peaks was also relatively longer.

**Figure 13:**
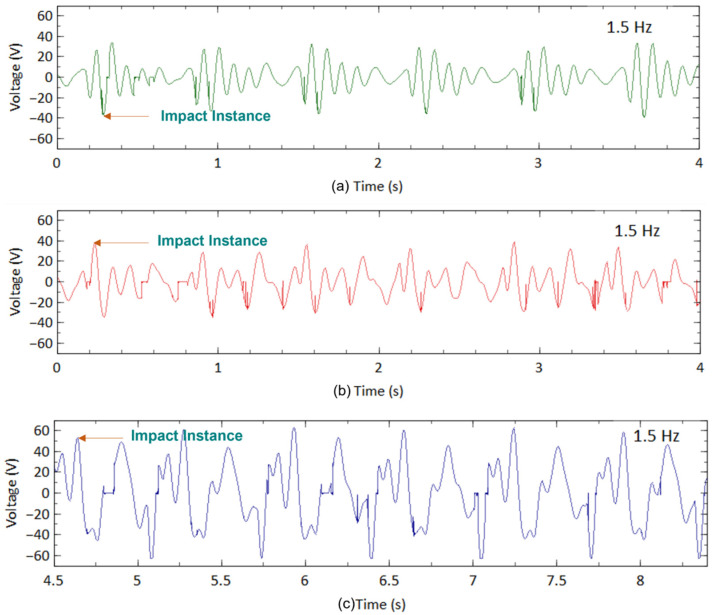
Voltage output of (**a**) CBH, (**b**) VBH, and (**c**) BBBH for human running motion (1.5 Hz).

Similarly, a running test was conducted to further investigate the proposed design’s performance. Figure 13a shows the voltage harvested by the CBH during running with an impact frequency of 1.5 Hz. The acceleration was maintained at approximately 0.45 g. For each design, four motion cycles were considered where six impacts were encountered. The highest output voltage generated by the CBH during running motion was 37 V, which was considerably higher than the walking motion. Once the impact was released, the output voltage decreased dramatically, similar to the walking and shaker test cases. Figure 13b represents the voltage output produced by VBH. The highest voltage obtained was nearly 40 V. In the remainder of the period, a minimum of 10 V was often achieved in one cycle. This was a notable improvement over the CBH.

In contrast, the BBBH (Figure 13c) showed a significant voltage improvement in running motion. In each cycle, the highest voltage achieved was 60 V. Between each impact, multiple voltage peaks ranging from 30 V to 60 V originated (further discussed in the next section). This was a vast improvement compared to the performance of CBH and VBH.

#### 3.2.2. Idle Time

To illustrate the idle time variation among the harvesters for human motion, a walking motion was considered. In the case of CBH, the peak voltage achieved was 20 V at each impact during a single motion cycle (Figure 12a). However, the voltage generated was heavily damped following each impact. Apart from the spike in voltage within a short period, approximately 0.5 s, or nearly half of the motion cycle, was idle or wasted. In contrast, if the peak voltage (20 V) of CBH was considered as the baseline, nearly 0.5 s after the impact instance, the voltage generated was reduced to 5 V, illustrating approximately a 75% voltage drop. In the next half-motion cycle, the voltage further reduced to 2 V, indicating a 90% reduction. The voltage generated during the idle time was remarkably smaller than the peak voltage. This relatively long idle time rendered the CBH rather inefficient. It is worth noting that this behavior was similar to the observation from the mechanical shaker test.

The peak voltage achieved for VBH (22 V, illustrated in Figure 12b) was slightly improved compared to CBH. For a fair comparison of idle time, if the same baseline (peak voltage of CBH) was considered for VBH, after 0.5 s of the impact, the voltage dropped to 10 V, emphasizing a 50% voltage reduction. During the next half cycle, the voltage slightly reduced to 7 V and persisted until the next impact. Thus, the voltage reduction at the end of the latter half-cycle of the motion was about 75%. Compared to CBH, this was a slight improvement.

Figure 12c depicts the voltage generation of BBBH during the walking motion. The peak voltage generated by BBBH was nearly 40 V, approximately twice that of CBH and VBH. Thus, if the same baseline (peak voltage of CBH) was considered for BBBH to maintain a fairground, the peak voltage achieved by BBBH at the impact instance was 200% compared to the baseline voltage. After 0.5 s from the impact, BBBH still produced a maximum of 30 V, about 150% of the baseline voltage. During the latter half of the motion cycle, the voltage varied between 15 V and 20 V, indicating a maximum of 25% reduction from the baseline voltage. This was deemed a significant improvement compared to the existing designs in idle time, and the observations were similar to the shaker test.

For running motion, a similar trend was noticed. However, the idle time in walking motion was longer than in running motion, most likely caused by the higher frequency and acceleration in the running along with the complexity of human motion. In addition to the peak voltage generated during the motion cycle, the number of voltage spikes that appeared was also different for each harvester. Table 9 summarizes the number of voltage peaks generated by each harvester under different motion types. The table summarizes the results of Figure 12 and Figure 13 in various forms. For instance, a higher number of voltage spikes exceeding the baseline voltage indicates that less time in a motion cycle is wasted (i.e., less idle time).

For this case, the number of voltage spikes achieved for walking motion was considered as an example. As above, the peak voltage (20 V) obtained for CBH was considered the baseline voltage. During a single motion cycle, the CBH was able to produce only one voltage peak. On the other hand, VBH managed to generate two voltage peaks during a single motion cycle. In the case of BBBH, six noticeable voltage peaks were in a single motion. This further certified the superior improvement of BBBH against CBH and VBH in terms of idle time. As stated earlier, reducing idle time could significantly influence the efficiency of a harvester. A similar comparison could be made for running motion. Since the frequency of running motion was considered as 1.5 Hz in this study, instead of a single motion cycle, two motion cycles had to be considered when assuming that three impacts were made during two motion cycles. The baseline voltage for running motion was 37 V. For simplicity, the results are presented in Table 9.

Even though the idle time was improved in BBBH among the other two designs, higher idle time was noticed for human motion compared to the shaker test due to the higher damping present in human motion. This could have been a result of the difficulty in the rigidity of fixing the PEH on the human leg, as muscles and skin could dampen the generated vibration.

#### 3.2.3. Harvested Power

**Figure 14:**
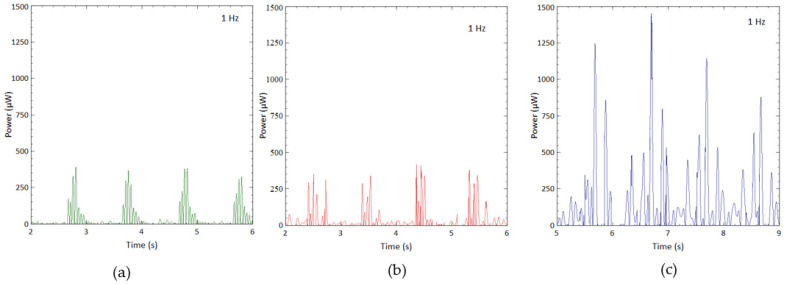
Power generated by (**a**) CBH, (**b**) VBH, and (**c**) BBBH for human walking (1 Hz).

**Figure 15:**
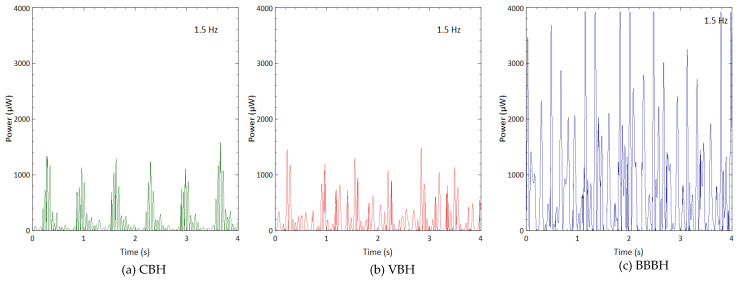
Power generated by (**a**) CBH, (**b**) VBH, and (**c**) BBBH for human running motion (1.5 Hz).

Similar to the shaker test, 1 MΩ resistance was used to represent the open-circuit condition. Figure 14 shows the power generated during normal walking, while Figure 15 shows the results obtained from running motion. For both scenarios, the CBH could produce only one power peak in one motion cycle with a magnitude of approximately 375 μW and 1400 μW for walking and running motions, respectively. On the other hand, the VBH produced slightly higher power peaks in a single motion cycle, achieving about 400 μW and 1500 μW for walking and running motions, respectively. This trend was very similar to the shaker test. Compared to CBH, VBH managed to produce a minimum of 50 μW throughout the period for walking and a minimum of 200 μW throughout the period of running. Once again, the BBBH harvested the highest amount of power, reaching nearly 1400 μW and 4000 μW (Figure 14c and Figure 15c) for walking and running motions, respectively. In contrast, BBBH generated a minimum of 150 μW and 400 μW for walking and running motions, respectively, throughout the period of interest.

#### 3.2.4. Average Output Power

**Figure 16:**
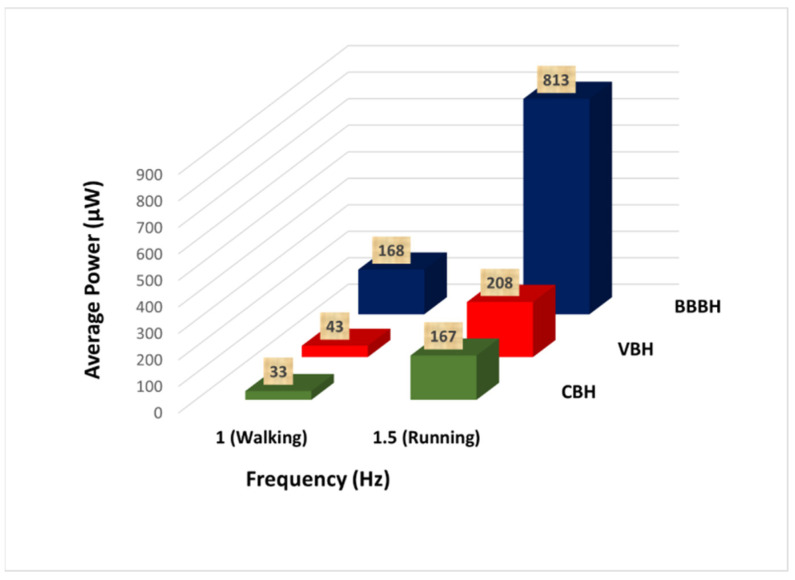
Comparison of average power generated by three different designs of harvesters for human motion.

The average output power generated for the human motion was calculated and is presented in Figure 16. For both walking and running motions, the average power output increased slightly from the CBH to the VBH and then considerably to the BBBH, highlighting the superior performance of the BBBH. The CBH and the VBH produced rather low average power for walking motion due to their relatively low output power and considerably long idle time. In contrast, the BBBH produced much higher output power with a higher number of power peaks and shorter idle time. Thus, the BBBH could produce a remarkably higher average power output of 168 μW compared to 33 μW and 43 μW of CBH and VBH, respectively.

For running motion, the CBH produced 167 μW of average output power, similar to the average power production of the BBBH during the walking motion. The VBH produced an average power of nearly 208 μW during running motion, slightly better than the CBH. On the other hand, the average output power produced by the BBBH was dramatically higher, achieving 813 μW. This outcome again confirmed the superiority of the proposed BBBH design in the ultralow-frequency range. The BBBH was also identified to perform the best during the running motion, as the average or highest output power generated was several orders higher than the other two designs.

With remarkably higher output voltage, output power, and average output power, as well as shorter idle time, the BBBH was identified to be the best performer among the three designs under all testing scenarios considered in this study.

#### 3.2.5. Performance Comparison with Existing Designs

In terms of average output power, a performance comparison was made between the proposed BBBH and selected designs for human motion applications available in the literature, as summarized in Table 10. All the harvester designs were attached to the human leg and tested during the human walking motion. The proposed design (BBBH) achieved 168 μW of average power compared to the VBH design (which existed in the literature), which generated 100 μW. In addition, the proposed BBBH showed superior power output compared to the other existing designs.

There are a few studies in the literature focusing on the development of low-power-consuming (<100 μW) wireless sensor nodes (WSNs) powered by energy harvesters for different applications [35,36]. The results obtained in this study prove that the proposed BBBH is applicable for powering such WSNs using ultralow-frequency vibration sources as an input with the aid of a proper rectifier circuit to stabilize, rectify, and amplify the voltage generated [9,37,38,39].

## 4. Conclusions

In this study, a novel bent branched beam PEH (BBBH) was proposed to improve the PEH’s energy generation capability from ultralow-frequency ambient vibration sources, such as human motion. A series of experimental and numerical activities were conducted to investigate the performance of the proposed design in terms of operating bandwidth, voltage generation, idle time, and power generation. Initially, the proposed BBBH was numerically studied and geometrically optimized. The BBBH was then fabricated and experimentally tested. Its performance was then compared with the conventional cantilever and a V-shaped beam harvester. Mechanical shaker tests and human motion tests (walking and running motions) were conducted to assess the feasibility of the proposed design in practical applications.

Three close resonance peaks (4.09 Hz, 8.72 Hz, and 10.56 Hz) were achieved by the proposed BBBH within the ultralow-frequency range (1 Hz to 10 Hz), indicating a widening operating bandwidth compared to the CBH and VBH designs. At its first resonance frequency, BBBH could achieve a peak voltage output of 44 V. Moreover, BBBH could perform remarkably at its second natural frequency, which was around 8 Hz (FEA 8.72 Hz), with a voltage output of 62 V. It was noted in the mode shape analysis, that both branches were vibrating in the same direction for the first, second, third, and fifth resonances which could help to generate maximum strain in MFC. The gaining of a maximum strain in MFC leads to the generation of a higher output voltage by enhancing the efficiency of the energy harvester.

Additionally, the BBBH showed considerable voltage and power generation improvement in all tests. The highest average power of 786 μW was obtained at 4 Hz during the shaker test and 813 μW during the human motion test (running). The idle time was also greatly reduced compared to the CBH (only 1/24 in the shaker test at 1 Hz under 1 g acceleration and 1/6 in the case of walking motion). These improvements are significant for a simple harvester design such as the BBBH, which operates without the aid of additional components, including excessive proof masses, magnets, and stoppers. The geometrical parameters of the harvester were selected as a proof of concept, and the harvester can be fabricated as a miniaturized device as required for practical human motion applications. With the aid of a rectifier circuit for power management, the proposed BBBH can potentially be used as a standalone energy provider for low-power-consuming devices such as WSNs and medical implants. For future work, focus can be placed on understanding the influence of multidirectional vibrations on the bent branches of the proposed harvester and developing a theoretical model to comparatively study the experimental results.

## Figures and Tables

**Table 1:** Material properties of aluminum beam and joint.

Parameters	Aluminum Beam	Joint
Materials	Aluminum alloy 6063-T5	Stainless steel 304
Elastic modulus (GPa)	68	190
Poisson’s ratio	0.33	0.26
Density (kg/m^3^)	2700	8100

**Table 2:** MFC actuator material properties.

Property	Young’s Modulus E_1_ (GPa)	Young’s Modulus E_2_ (GPa)	Poisson’s Ratio V_12_	Shear Modulus G_12_ (GPa)	Piezoelectric Charge Constants d_33_ (pC/N)	Piezoelectric Charge Constants d_31_ (pC/N)	Active Area Density (g/cm^3^)
Value	30.336	15.857	0.31	5.515	4.6 × 10^2^	−2.1 × 10^2^	5.44

**Table 3:** First six NF of main cantilever beam with varying length (width = 20 mm).

Design	Length (mm)	NF (Hz)					
		1	2	3	4	5	6
L1	75	30.43	160.11	317.55	870.29	1686.80	2511.40
L2	100	18.68	124.60	209.00	543.28	833.72	1947.50
L3	150	9.54	91.69	115.67	282.77	346.57	793.72
L4	200	5.39	65.47	69.27	168.85	186.32	388.08
L5	250	4.15	47.98	62.57	128.83	138.92	276.79

**Table 4:** First six NFs of a main cantilever beam with varying width (length = 200 mm).

Design	Width (mm)	NF (Hz)					
		1	2	3	4	5	6
W1	20	5.39	65.47	69.27	168.85	186.32	388.08
W2	25	6.57	73.83	83.43	212.28	236.63	437.95
W3	30	7.09	75.07	89.40	218.52	293.88	446.65

**Table 5:** First six NFs of each PEH design.

Design	NF (Hz)					
	1	2	3	4	5	6
CBH	5.39	65.47	69.27	168.85	186.32	388.08
VBH	4.50	11.80	14.24	72.09	130.68	132.43
BBBH	4.09	8.72	10.56	14.47	55.71	90.63

**Table 6:** Highest stress and strain achieved by various PEH under static FEM.

PEH Types	Parameters	Highest Recorded Values
CBH	Strain (m/m)	1.28 × 10^−5^
	Stress (Pa)	8.73 × 10^5^
VBH	Strain (m/m)	1.29 × 10^−5^
	Stress (Pa)	8.77 × 10^5^
BBBH	Strain (m/m)	1.43 × 10^−5^
	Stress (Pa)	9.74 × 10^5^

**Table 7:** Variables engaged in shaker and human motion tests.

Tests	Variables	
	Acceleration (g)	Frequency (Hz)
Mechanical Shaker Test	1 and 2	1, 2, 3, 4, and 5
Human Motion Test	0.2 and 0.45	1 and 1.5

**Table 8:** Number of peaks obtained for different frequencies within one motion cycle under 1 g.

Acceleration	Frequency	Harvester Design	Baseline Voltage	The Number of Peaks Reached the Baseline Voltage
1 g	1 Hz	CBH	10 V (peak voltage of CBH)	1
		VBH		6
		BBBH		24
	2 Hz	CBH	13 V (peak voltage of CBH)	2
		VBH		8
		BBBH		24
	3 Hz	CBH	14 V (peak voltage of CBH)	3
		VBH		9
		BBBH		24
	4 Hz	CBH	16 V (peak voltage of VBH)	16
		VBH		8
		BBBH		24
	5 Hz	CBH	8 V (peak voltage of BBBH)	20
		VBH		10
		BBBH		5

**Table 9:** The number of voltage peaks obtained in one motion cycle for walking and running motion.

**Motion Type**	**Harvester Design**	**Number of Peaks Reaching Baseline Voltage (20 V) in One Cycle**
Walking (1 Hz)	CBH	1
	VBH	2
	BBBH	6
**Motion type**	**Harvester design**	**Number of peaks reaching baseline voltage (37 V) in two cycles**
Running (1.5 Hz)	CBH	3
	VBH	5
	BBBH	15

**Table 10:** Performance comparison of the BBBH with existing designs.

Reference	Descriptions	Types of Piezoelectric Materials	Speed/ Frequency	Motion Type	Average Output Power
Li, 2010 [27]	PEH with curved L-shaped proof mass	PZT (T215-H4-103Y)	4 km/h	Walking	49 μW
Fan, 2017 [32]	A shoe mounted PEH	PZT-5H	2 km/h	Walking	30 μW
Izadgoshasb, 2018 [25]	PEH with cantilever beam	MFC (M2814-P2)	6 km/h	Walking	N.A.
Izadgoshasb, 2019 [26]	PEH with a double-pendulum system	MFC (M2814-P2)	1 Hz	Walking	36 μW
Jiang, 2020 [23]	V-shaped bent beam harvester	PZT-5H	1 Hz	Feet stamping	100 μW
Nastro, 2022 [33]	Wearable Ball-Impact PEH	PZT (RS-pro 285–784)	4 Hz	Wrist rotations	9.65 µW
Wang, 2022 [34]	A multi-folded-beam PEH	PZT-5H	9.2 Hz	Running	29 μW
Proposed BBBH	Bent branched beam PEH	MFC (M2814-P2)	2 km/h	Walking	168 μW

## Data Availability

Data could be provided upon a request to the corresponding author.

## References

[1] Maamer B., Boughamoura A., El-Bab A.M.F., Francis L.A., Tounsi F. (2019). A review on design improvements and techniques for mechanical energy harvesting using piezoelectric and electromagnetic schemes. Energy Convers. Manag..

[2] Erturk A., Inman D.J. (2011). Piezoelectric Energy Harvesting.

[3] Kathpalia B., Tan D., Stern I., Erturk A. (2017). An experimentally validated model for geometrically nonlinear plucking-based frequency up-conversion in energy harvesting. Smart Mater. Struct..

[4] Yang Z., Zhou S., Zu J., Inman D. (2018). High-performance piezoelectric energy harvesters and their applications. Joule.

[5] Li Z., Yang Z., Naguib H.E. (2020). Introducing revolute joints into piezoelectric energy harvesters. Energy.

[6] Shan X., Tian H., Chen D., Xie T. (2019). A curved panel energy harvester for aeroelastic vibration. Appl. Energy.

[7] Yang Z., Wang Y.Q., Zuo L., Zu J. (2017). Introducing arc-shaped piezoelectric elements into energy harvesters. Energy Convers. Manag..

[8] Sun R., Li Q., Yao J., Scarpa F., Rossiter J. (2020). Tunable, multi-modal, and multi-directional vibration energy harvester based on three-dimensional architected metastructures. Appl. Energy.

[9] Edla M., Lim Y.Y., Deguchi M., Padilla R.V. A Novel Discontinuous Mode Piezoelectric Energy Harvesting Circuit for Low-Voltage Applications. Proceedings of the 2021 31st Australasian Universities Power Engineering Conference (AUPEC).

[10] Abohamer M., Awrejcewicz J., Amer T. (2023). Modeling of the vibration and stability of a dynamical system coupled with an energy harvesting device. Alex. Eng. J..

[11] Abohamer M.K., Awrejcewicz J., Starosta R., Amer T.S., Bek M.A. (2021). Influence of the motion of a spring pendulum on energy-harvesting devices. Appl. Sci..

[12] Lin Z., Al Ba’ba’a H., Tol S. (2021). Piezoelectric metastructures for simultaneous broadband energy harvesting and vibration suppression of traveling waves. Smart Mater. Struct..

[13] Kouritem S.A., Al-Moghazy M.A., Noori M., Altabey W.A. (2022). Mass tuning technique for a broadband piezoelectric energy harvester array. Mech. Syst. Signal Process..

[14] Fan K., Liu S., Liu H., Zhu Y., Wang W., Zhang D. (2018). Scavenging energy from ultra-low frequency mechanical excitations through a bi-directional hybrid energy harvester. Appl. Energy.

[15] Khalid S., Raouf I., Khan A., Kim N., Kim H.S. (2019). A review of human-powered energy harvesting for smart electronics: Recent progress and challenges. Int. J. Precis. Eng. Manuf.-Green Technol..

[16] Izadgoshasb I., Lim Y.Y., Vasquez Padilla R., Sedighi M., Novak J.P. (2019). Performance enhancement of a multiresonant piezoelectric energy harvester for low frequency vibrations. Energies.

[17] Wu H., Tang L., Yang Y., Soh C.K. (2013). A novel two-degrees-of-freedom piezoelectric energy harvester. J. Intell. Mater. Syst. Struct..

[18] Zhang G., Hu J. (2014). A branched beam-based vibration energy harvester. J. Electron. Mater..

[19] Upadrashta D., Yang Y. (2018). Trident-shaped multimodal piezoelectric energy harvester. J. Aerosp. Eng..

[20] Zhao Y., Qin Y., Guo L., Tang B. (2018). Modeling and experiment of a v-shaped piezoelectric energy harvester. Shock. Vib..

[21] Erturk A., Renno J.M., Inman D.J. (2009). Modeling of piezoelectric energy harvesting from an L-shaped beam-mass structure with an application to UAVs. J. Intell. Mater. Syst. Struct..

[22] Su W.-J., Zu J.W. Modeling of V-shaped beam-mass piezoelectric energy harvester: Impact of the angle between the beams. Proceedings of the ASME International Mechanical Engineering Congress and Exposition.

[23] Jiang W., Wang L., Zhao L., Luo G., Yang P., Ning S., Lu D., Lin Q. (2021). Modeling and design of V-shaped piezoelectric vibration energy harvester with stopper for low-frequency broadband and shock excitation. Sens. Actuators A Phys..

[24] Li X., Upadrashta D., Yu K., Yang Y. (2019). Analytical modeling and validation of multi-mode piezoelectric energy harvester. Mech. Syst. Signal Process..

[25] Izadgoshasb I., Lim Y.Y., Lake N., Tang L., Padilla R.V., Kashiwao T. (2018). Optimizing orientation of piezoelectric cantilever beam for harvesting energy from human walking. Energy Convers. Manag..

[26] Izadgoshasb I., Lim Y.Y., Tang L., Padilla R.V., Tang Z.S., Sedighi M. (2019). Improving efficiency of piezoelectric based energy harvesting from human motions using double pendulum system. Energy Convers. Manag..

[27] Li W.G., He S., Yu S. (2009). Improving power density of a cantilever piezoelectric power harvester through a curved L-shaped proof mass. IEEE Trans. Ind. Electron..

[28] Camilloni E., DeMaso-Gentile G., Scavongelli C., Orcioni S., Conti M. (2016). Piezoelectric energy harvesting on running shoes. Mobile Networks for Biometric Data Analysis.

[29] Wei S., Hu H., He S. (2013). Modeling and experimental investigation of an impact-driven piezoelectric energy harvester from human motion. Smart Mater. Struct..

[30] Moro L., Benasciutti D. (2010). Harvested power and sensitivity analysis of vibrating shoe-mounted piezoelectric cantilevers. Smart Mater. Struct..

[31] Southgate D., Childs P., Bull A. (2016). Sports Innovation, Technology and Research.

[32] Fan K., Liu Z., Liu H., Wang L., Zhu Y., Yu B. (2017). Scavenging energy from human walking through a shoe-mounted piezoelectric harvester. Appl. Phys. Lett..

[33] Nastro A., Pienazza N., Baù M., Aceti P., Rouvala M., Ardito R., Ferrari M., Corigliano A., Ferrari V. (2022). Wearable Ball-Impact Piezoelectric Multi-Converters for Low-Frequency Energy Harvesting from Human Motion. Sensors.

[34] Wang J.-X., Li J.-C., Su W.-B., Zhao X., Wang C.-M. (2022). A multi-folded-beam piezoelectric energy harvester for wideband energy harvesting under ultra-low harmonic acceleration. Energy Rep..

[35] Jang J., Berdy D., Lee J., Peroulis D., Jung B. (2012). A wireless condition monitoring system powered by a sub-100/spl mu/W vibration energy harvester. IEEE Trans. Circuits Syst. I Regul. Pap..

[36] Zhang F., Zhang Y., Silver J., Shakhsheer Y., Nagaraju M., Klinefelter A., Pandey J., Boley J., Carlson E., Shrivastava A. A batteryless 19μW MICS/ISM-band energy harvesting body area sensor node SoC. Proceedings of the 2012 IEEE International Solid-State Circuits Conference.

[37] Edla M., Lim Y.Y., Vasquez Padilla R., Deguchi M. (2021). An Improved Rectifier Circuit for Piezoelectric Energy Harvesting from Human Motion. Appl. Sci..

[38] Edla M., Lim Y.Y., Deguchi M., Padilla R.V., Izadgoshasb I. (2020). An improved self-Powered H-bridge circuit for voltage rectification of piezoelectric energy harvesting system. IEEE J. Electron Devices Soc..

[39] Edla M., Mikio D., Izadgoshasb I., Mahmud M.A.P., Kouzani A.Z. (2022). Self-powered boost-converter for power optimisation and piezo garden lights. Smart Mater. Struct..

